# Exploration of the diagnostic and prognostic roles of decreased autoantibodies in lung cancer

**DOI:** 10.3389/fimmu.2025.1538071

**Published:** 2025-01-30

**Authors:** Ying Ye, Yi Huang, Jianbo Pan

**Affiliations:** ^1^ Basic Medicine Research and Innovation Center for Novel Target and Therapeutic Intervention, Ministry of Education, College of Pharmacy, Chongqing Medical University, Chongqing, China; ^2^ Department of Cardiothoracic Surgery, The Second Affiliated Hospital of Chongqing Medical University, Chongqing, China; ^3^ Shengli Clinical Medical College, Fujian Medical University, Fuzhou, Fujian, China; ^4^ Department of Clinical Laboratory, Fujian Provincial Hospital, Fuzhou, Fujian, China; ^5^ Precision Medicine Center, The Second Affiliated Hospital of Chongqing Medical University, Chongqing, China

**Keywords:** TAA autoantibodies, decreased autoantibodies, early-stage lung cancer, diagnostic models, novel tumor markers

## Abstract

**Introduction:**

Tumor-associated antigens (TAA) are proteins expressed during the growth and development of tumor cells, and TAA autoantibodies (TAAbs) can be detected in the serum of lung cancer patients, which can be utilized in the early screening of lung cancer. Almost all the TAAbs applied for diagnosis are those elevated, however, there are still large numbers of autoantibodies detected to decrease in tumor serums, and their functions were rarely known. Diagnosing malignant small lung nodules (≤3cm) in CT scans remains a challenge in clinical practice.

**Methods:**

In this study, we applied the HuProt array and the bioinformatics analysis to assess the diagnostic values of the decreased autoantibodies in lung cancers.

**Results:**

In total, 15 types of decreased autoantibodies were identified, and 6 of them were constructed into a predictive model for early lung cancer, reaching a sensitivity of 76.19% and a specificity of 55.74%. We combined with 4 elevated TAAbs, the sensitivity and the specificity of the 10-marker model can attain 80.0% and 87.0%, respectively, which is higher than that of the commonly used 7-TAAbs model in diagnosis for early-stage lung cancer. Moreover, 5 of the decreased autoantibodies can also be applied for supervising bone metastasis in lung adenocarcinoma. A follow-up process for 13 patients diagnosed with early-stage lung cancer revealed that 10 of the 15 decreased autoantibodies would recover to a higher level after the tumor was resected. Bioinformatic analysis indicated that the 15 biomarkers were strongly correlated with the prognosis of lung cancer patients.

**Conclusion:**

We confirmed the importance of the decreased autoantibodies in lung cancer, providing new diagnostic and therapeutic strategies.

## Introduction

Lung cancer is the most common malignant tumor in the world, and its incidence and mortality rates have been increasing over the years. In 2020, it is estimated about 2.2 million new cases of lung cancer and 1.8 million lung cancer-related deaths, and globally, lung cancer is the leading cause of cancer death in men and the second leading cause of cancer death in women (1). Despite advances in treatment methods in recent years, the prognosis of lung cancer remains poor, with an overall 5-year survival rate of less than 20%, even under a 5% survival rate in advanced tumor stages (2). The 5-year survival rate of early-stage lung cancer can be increased by 20% to 30% through surgical treatment, and the survival rate of stage IA patients can even be improved to more than 90% by timely treatment (3). Thus, early detection and treatment are crucial to reduce the mortality of lung cancer.

With the popularization of thin-slice CT examination, increasing numbers of small pulmonary nodules could be recognized. Studies indicate that lung nodules occur in about 50% of patients undergoing chest CT, and approximately 95% of them are benign lesions (4). In clinical practice, stage IA lung cancer appears as a nodule ≤3 cm in diameter, ground-glass, or solid-like, which is difficult to distinguish from benign lesions, especially from those with chronic pulmonary inflammation. Regular follow-up is often used to dynamically observe the changes of nodules through CT images, but this method is still not accurate enough, it may increase the radiation dose of patients (5). Another commonly used method for clarifying the nature of the pulmonary nodules is percutaneous puncture biopsy, however, biopsies may lead to complications such as pneumorrhagia and pneumothorax, so most patients are reluctant to undergo invasive procedures (6). Therefore, the diagnosis of benign and malignant pulmonary nodules has become another major clinical challenge.

As a non-invasive examination method, liquid biopsy has been widely recognized in the early screening, diagnosis, and follow-up of lung cancer (7). Biomarker-based detection methods have gradually improved the accuracy of early lung cancer diagnosis. Traditional serum tumor markers, including carcinoembryonic antigen (CEA), neuron-specific enolase (NSE), human cell keratin 21-1 fragment (CYFRA21-1), and some other types, are routinely used for lung tumor screening (8). However, as the markers are not specific to lung cancer, they are commonly used for evaluating therapeutic effects and monitoring recurrence (9). CEA levels are high in lung adenocarcinomas (LUAD) but low in other types of lung cancer, therefore, CEA has limited diagnostic value in early-stage lung cancer of non-adenocarcinoma types (10). NSE is mainly used for the diagnosis and monitoring of small cell lung cancer (SCLC), it has less diagnostic value for early-stage non-small cell lung cancer (NSCLC) (11). CYFRA21-1 levels are high in squamous cell carcinoma (LUSC), but the diagnostic value of CYFRA21-1 is limited in early-stage lung cancer of non-squamous cell carcinoma types (12).

Thus, there is an urgent need to find a highly sensitive and specific tool with little adverse effects that can be applied to lung cancer screening for the general public, to improve the diagnosis and treatment of early-stage lung cancer and its survival rate.

Recently, the detection of tumor-related antigens autoantibodies (TAAbs) is one of the promising applications for cancer early diagnosis (13). TAAbs are abnormal exposure of antigens from cancerous cells during tumor development, which stimulates the activation and proliferation of B cells, expanding a large number of antibodies against tumor antigens (14). These autoantibodies are highly stable proteins that can be detected months to years before the onset of clinical symptoms in patients and persist for a long period after the disappearance of TAs (15). In prior studies, the most concerned autoantibodies are those that are elevated in tumor serum samples, and for instance, a predictive panel of three autoantibodies has been established (16). Moreover, the commonly recognized 7-TAAbs (including p53, PGP9.5, SOX2, GAGE7, GBU4-5, MAGEA1, and CAGE) diagnostic model all consist of markers elevated in tumor serum (17). However, little is known about the decreased autoantibodies in tumor patients. In addition, 7-TAAbs tend to have high diagnostic efficiency in advanced lung cancer, but in patients with early-stage disease, the sensitivity of 7-TAAbs is usually lower, less than 30% (18). Therefore, identifying new autoantibody-associated markers to improve the diagnostic efficiency of early-stage lung cancer is essential.

In this study, we identified a particular group of autoantibodies by applying the protein array-based approach. These autoantibodies were identified as significantly decreased in the serum of patients with malignant pulmonary nodules compared to those with benign lung lesions, and some of them were found to return high levels after surgery in malignant pulmonary nodules patients. Moreover, a few autoantibodies were discovered specifically decreased in the serum of LUAD patients accompanied by bone metastasis.

## Materials and methods

This study is conceived as re-analysis research based on our previous data. The enrollment of subjects, the collection of serum samples, and the ethical approval were detailed and described in our prior paper (Approved by the Ethics Committee of Fujian Provincial Hospital, NO. K2014-01-002) (16).

### HuProt array preparation

The HuProt array provided by CDI Laboratories, Inc., which covers over 75% of the human proteome, containing 20,240 unique human full-length proteins, was employed for our preliminary screening as described previously. In the preliminary screening phase, the serums from 80 lung cancer (LC) patients and 20 healthy subjects were analyzed by the HuProt array. The LC group contains 24 adenocarcinomas, 23 squamous carcinomas, 13 large-cell lung cancers, and 20 small-cell lung cancers. The healthy serum samples were collected from those persons who undergo regular annual health examinations and are without evidence of any malignant diseases. According to the screening criteria, 199 candidate protein biomarkers were further analyzed in our LC Focused Arrays as our previous study mentioned (16).

### Data collection

We aimed to explore the diagnostic values of these candidate autoantibodies in distinguishing early-stage LCs from lung benign lesions (LBLs), therefore, 101 LBL and 105 early-stage LC patients were enrolled (**Table 1**). The LC group included 75 stage IA NSCLCs and 30 SCLCs with tumor long diameter ≤ 3cm). The LBL cohort contains 55 patients with pneumonia, 26 with chronic obstructive pulmonary disease (COPD), and 20 with pulmonary tuberculosis (TB), which exhibited pulmonary shadows or nodes (long diameter ≤ 3cm) in the primary diagnostic CT scan. The benign lesions were confirmed by the shrinkage or disappearance of mass in the subsequent CT scan after treatment with antibiotic/antituberculosis therapies. The malignant diagnoses for early LC patients were ascertained by histopathology after surgical resection.

**Table 1:** The characteristics between early LC patients and LBL subjects.

Variables	Early LC(N=105)	LBL(N=101)	*P*-value
**Age**	60.92 ± 10.16	61.07 ± 8.78	0.913
**Sex**			0.999
Male	79 (68.70%)	69 (68.32%)	
Female	36 (31.30%)	32 (31.68%)	
**Smoking**			0.1877
never	18 (17.14%)	28 (27.72%)	
<20 packyears	26 (24.76%)	21 (20.79%)	
≥20 packyears	61 (58.10%)	52 (51.49%)	
**Tumor Type**			
LUAD	43 (40.95%)		
LUSC	32 (30.48%)		
SCLC	30 (28.57%)		

Moreover, another 305 advanced LCs (stage II~IV), 35 colorectal cancers, 61 hepatic carcinomas, 27 cervical cancers, 48 esophageal cancers, 51 gastric cancers, 14 ovarian cancers, and 15 systemic lupus erythematosus (SLEs) were enrolled to validate the diagnostic specificity of the decreased autoantibodies in early-stage LCs.

5 milliliters of venous blood was collected into a blood tube with diatomite coagulant from each patient and then centrifuged for 10 minutes at 4000 rpm at room temperature within 4 h after collection. After that, the serums were transferred to the 1.5 ml EP tubes and then stored at -80°C until use.

To understand the specific functions of the identified autoantibodies, the corresponding mRNAs and proteins were further studied by applying the GEO database (GSE60052 and GSE135304), TCGA database, cBioPortal database, and CPTAC (Clinical Proteomic Tumor Analysis Consortium) database. The mRNA and protein levels and the clinical data were utilized for bioinformatic analysis. The immune infiltration analysis was accomplished by the online tool TIMER 2.0 (http://timer.comp-genomics.org/).

**Figure 1:**
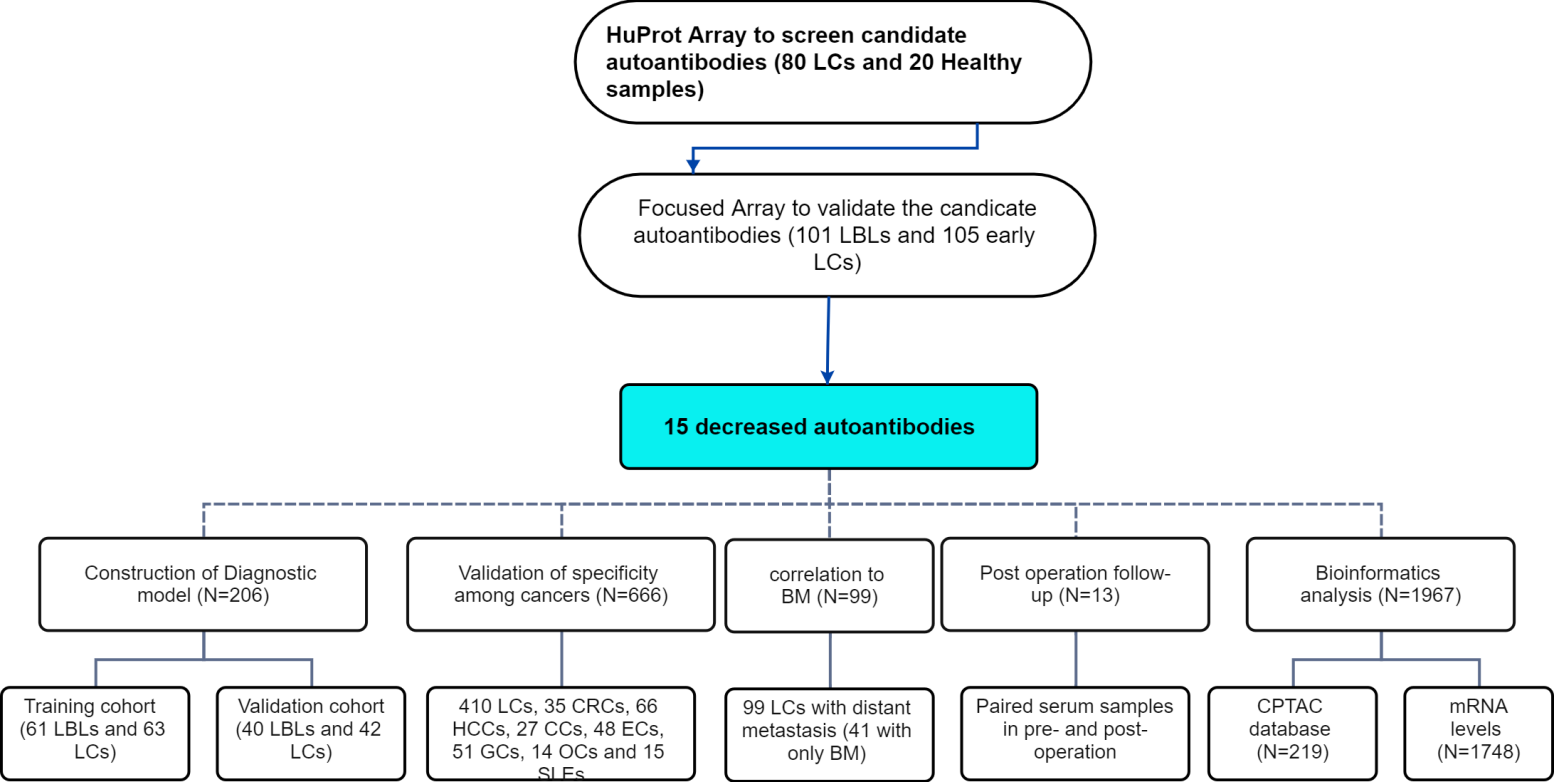
The graphical workflow maps of the study.

The analysis process of this study is shown in **Figure 1**.

### Statistical analysis

The statistical analyses and the plotting methods were all accomplished with R software (version 4.1.1) and GraphPad (version 9.0). When comparing the protein and gene levels, once the data followed a normal distribution, the Student’s t-test was utilized, otherwise the Wilcoxon rank-sum test was applied. We applied the multiple logistic regression analysis to construct our predictive models, and the best model was determined according to the maximums of sensitivity and specificity. The Kaplan–Meier curve analysis in the CPTAC database was accomplished by the “survival” and “survminer” R packages, and the Kaplan–Meier curve for each mRNA was obtained from the “Kaplan-Meier Plotter” online tool (https://www.kmplot.com/analysis/).

## Results

### Identification of the down-regulated autoantibodies in early LCs

**Figure 2:**
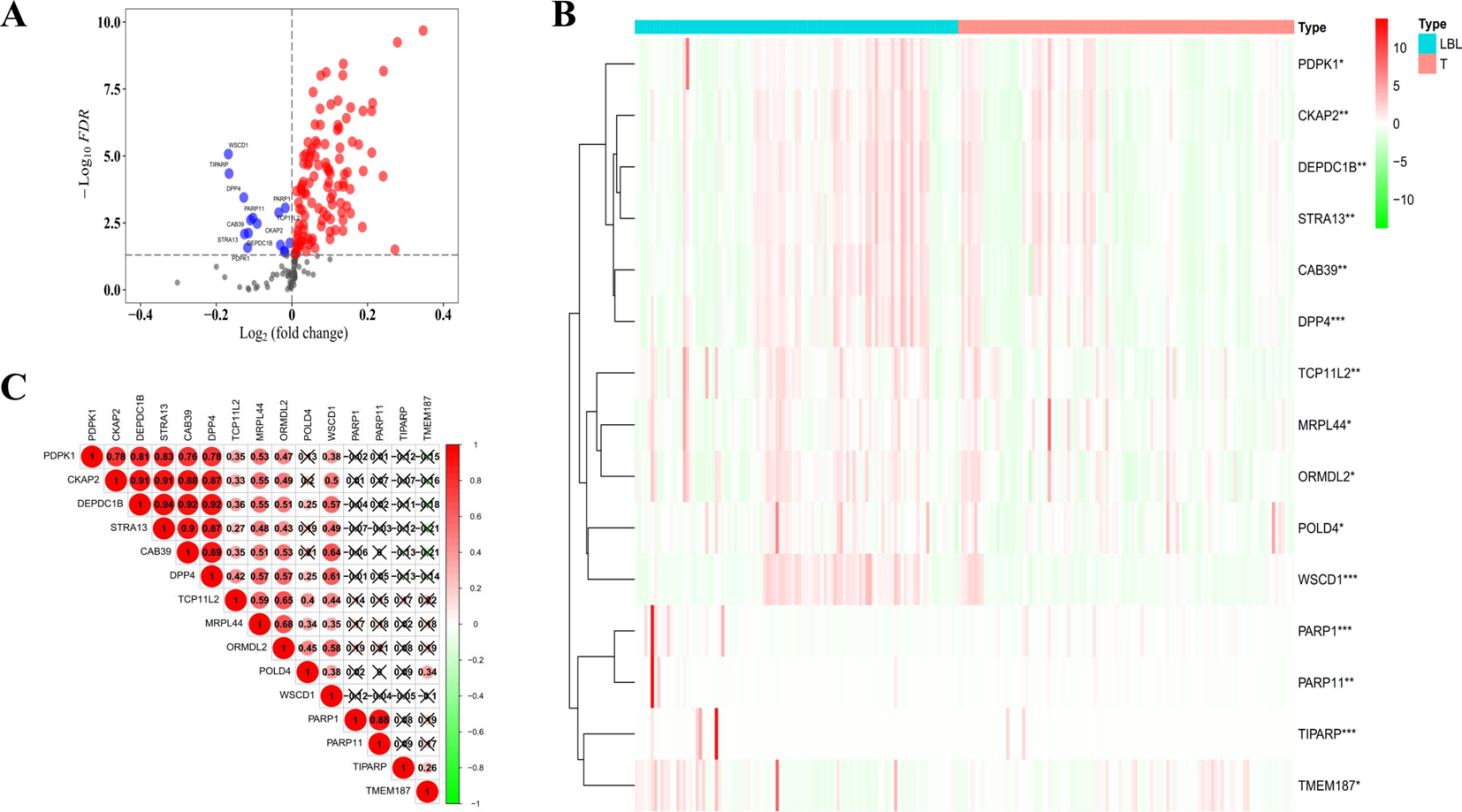
Down-regulated autoantibodies in early LC. **(A)** volcano plot for the up-(red) and down-(blue) regulated autoantibodies in tumor **(B)** heatmaps for the 15 candidate autoantibodies in LBLs (blue) and tumor (red), *P < 0.05; **P < 0.01; ***P < 0.001) **(C)** spearman correlation analysis for the 15 autoantibodies (red, positive correlations; green, negative correlations).

By applying the LC Focused Arrays, we compared the levels of 199 candidate protein biomarkers between the 101 LBL and 105 early LC (Stage IA) subjects, and finally, 133 differentially expressed proteins were detected, with the criteria of *P <*0.05. Among them, 118 were enriched, while the other 15 autoantibodies (CAB39, CKAP2, DEPDC1B, DPP4, MRPL44, ORMDL2, PARP1, PARP11, PDPK1, POLD4, STRA13, TCP11L2, TIPARP, TMEM187, WSCD1) were decreased in the early LC group (**Figure 2A**), and the protein level heatmap of each decreased autoantibody was shown in **Figure 2B**. To better study the correlations among these decreased autoantibodies, a Spearman correlation analysis was performed (Red: positive correlation, Green: negative correlation, **Figure 2C**), and the results indicated that most of them were strongly positively related, other than the 4 elements (PARP1, PARP11, TIPARP, and TMEM187).

### Exploration of the diagnostic values of the decreased autoantibodies in early LCs

**Figure 3:**
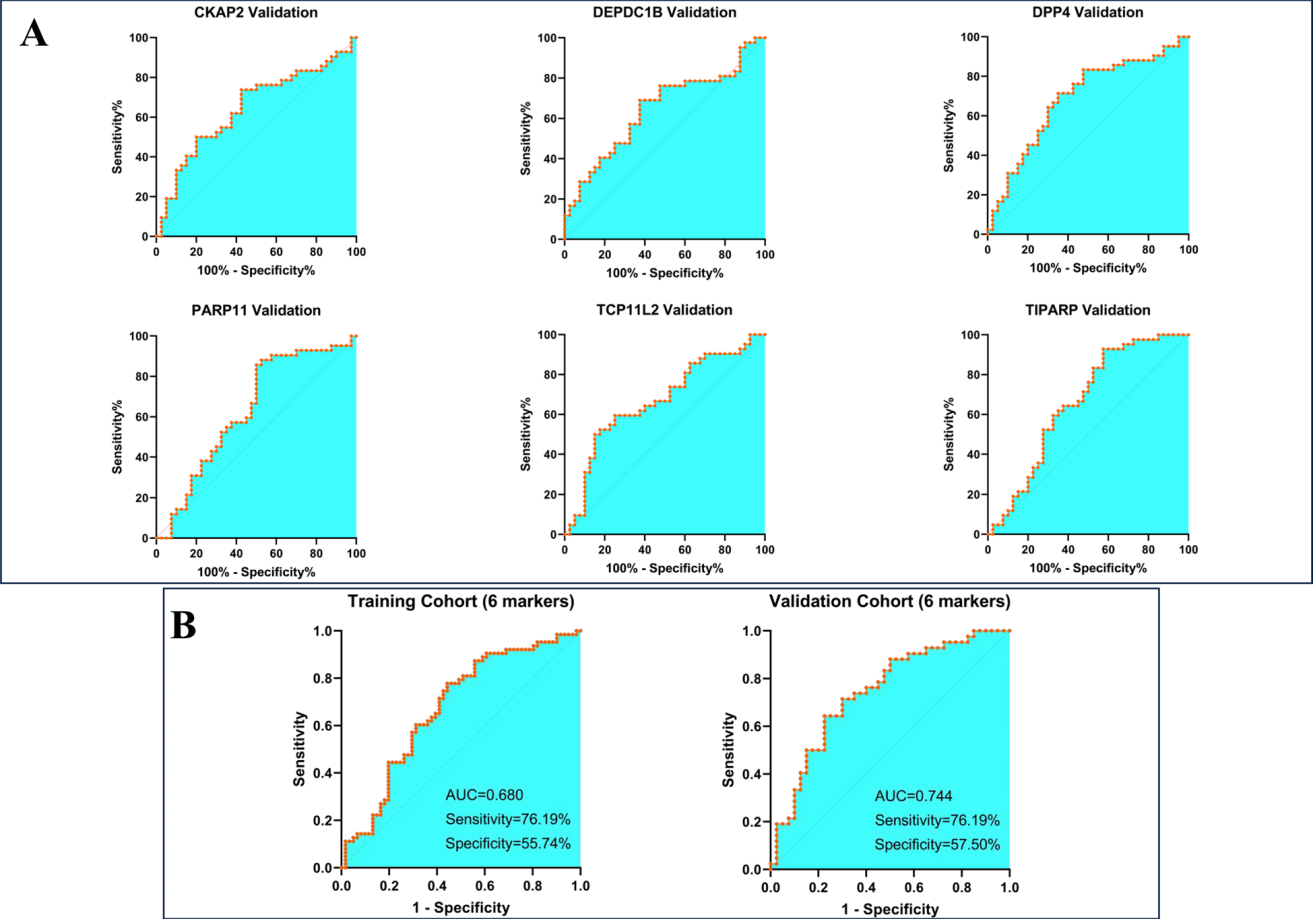
Construction and validation of a 6-maker diagnostic model. **(A)** Validation of each maker in the diagnostic model **(B)** the diagnostic efficacy of the model in training and validation cohort.

In this phase, 101 LBLs and 105 early LCs were respectively randomly divided into new cohorts according to the ratio of 6:4 (utilizing the random number table method), thus a training cohort containing 61 LBLs and 63 LCs, while a validation cohort including the rest subjects was established. We first evaluated the diagnostic value of each candidate autoantibody by applying the receiver operating characteristic (ROC) curve. The best cut-off value for each protein was calculated by the following criteria: with specificity≥90%, P-value<0.05, meanwhile with the maximum Youden index in the training cohort. The ROC curves of the 15 proteins were presented in **Supplementary Figure S1**, and the area under the curve (AUC) values of the proteins ranged from 0.56 to 0.69. We then employed the validation cohort for verification, and the results confirmed the diagnostic value of these autoantibodies (**Table 2**). To establish combinatorial biomarker panels for better performance of predictive ability, the multiple logistic regression analysis was utilized for screening the best combination. Finally, 6 markers comprised of CKAP2, DEPDC1B, DPP4, PARP11, TCP11L2, and TIPARP were retained according to the optimal logistic regression model in the training cohort, and their predictive efficacies in the validation cohort were shown as ROC curves in **Figure 3A**. The 6-marker model reached a high sensitivity of 76.19% while at a relatively low specificity of 55.74% in the training cohort, and the results were similar in the validation group (**Figure 3B**).

**Table 2:** The diagnostic value of each decreased autoantibody.

Proteins	Training cohort				Validation cohort		
	Cut-off	AUC	Sensitivity	Specificity	AUC	Sensitivity	Specificity
CAB39	1.295	0.635	20.63%	90.16%	0.610	19.05%	80.00%
CKAP2	1.294	0.644	28.57%	90.16%	0.575	21.43%	80.00%
DEPDC1B	1.334	0.640	19.05%	93.44%	0.563	19.05%	80.00%
DPP4	1.205	0.674	14.29%	91.80%	0.594	7.14%	87.50%
MRPL44	1.099	0.610	14.29%	90.16%	0.560	19.05%	92.50%
ORMDL2	1.062	0.610	25.40%	90.16%	0.571	21.43%	80.00%
PARP1	1.096	0.625	20.63%	90.00%	0.639	35.71%	80.00%
PARP11	1.208	0.626	12.70%	90.00%	0.618	28.57%	82.50%
PDPK1	1.512	0.624	17.46%	91.80%	0.541	14.29%	92.50%
POLD4	1.010	0.560	15.87%	91.80%	0.631	21.43%	90.00%
STRA13	1.534	0.638	28.57%	91.80%	0.562	21.43%	85.00%
TCP11L2	1.025	0.610	20.63%	90.16%	0.657	33.33%	90.00%
TIPARP	1.076	0.689	17.46%	90.16%	0.627	26.19%	80.00%
TMEM187	1.255	0.602	7.94%	90.16%	0.586	9.52%	85.00%
WSCD1	1.035	0.674	12.70%	90.16%	0.689	26.19%	90.00%

### Development of a 10-marker predictive model of early LCs

**Figure 4:**
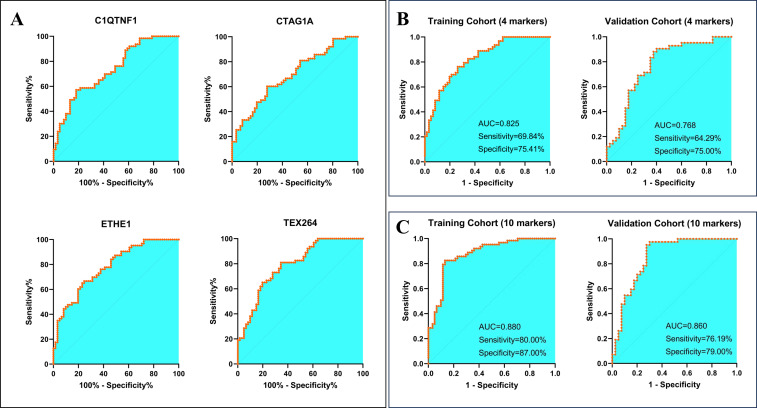
Construction of a diagnostic model in combination with increased autoantibodies. **(A)** the diagnostic efficacy of the 4 candidates increased autoantibodies in the training cohort **(B)** construction and validation of a 4-marker diagnostic model **(C)** establishment of a 10-marker diagnostic model.

As the specificity of the 6 decreased marker model is unsatisfactory, we determined to combine those increased markers to test whether the specificity could be improved. In our previous study, 8 markers (P53, ETHE1, CTAG1A, C1QTNF1, TEX264, CLDN2, NSG1, and HRAS) which were enriched in the serum of lung cancer patients were identified (16), among them, 3 markers (P53, ETHE1 and HRAS) were proved to own strongly predictive ability in distinguishing healthy population (including parts of LBLs) and stage I~II lung cancer patients. In this study, we proposed to make certain diagnoses of those patients with pulmonary nodules (all LBLs vs. stage IA lung cancer). We re-evaluated the predictive efficacies of these 8 markers and found that 4 markers (ETHE1, CTAG1A, C1QTNF1, and TEX264) revealed favorable predictive abilities in the training and validation cohort (**Figure 4A** and **Table 3**). The combination of 4 markers showed 69.84% sensitivity and 75.41% specificity in the training cohort, while 64.29% sensitivity and 75.00% specificity in the validation cohort (**Figure 4B**). It illustrated that these increased markers provided higher specificity, while the decreased markers displayed more sensitivity for predicting models. Thus, we constructed a new model that includes the 6 decreased markers and the 4 evaluated proteins and found that the 10-marker model can reach a sensitivity of 80.00%, and a specificity of 87.00% in the training group, while with 76.19% sensitivity and 79.00% specificity in the validation cohort (**Figure 4C**).

**Table 3:** The diagnostic value of each candidate increased autoantibody.

Proteins	Training cohort				Validation cohort		
	Cut-off	AUC	Sensitivity	Specificity	AUC	Sensitivity	Specificity
ETHE1	1.900	0.783	33.33%	91.80%	0.710	33.33%	90.00%
C1QTNF1	1.715	0.735	22.22%	96.72%	0.668	19.05%	90.00%
CTAG1A	1.187	0.689	22.22%	96.72%	0.629	21.43%	90.00%
TEX264	2.067	0.759	26.98%	91.80%	0.727	28.57%	90.00%

### The specificity of decreased autoantibodies among cancers

**Figure 5:**
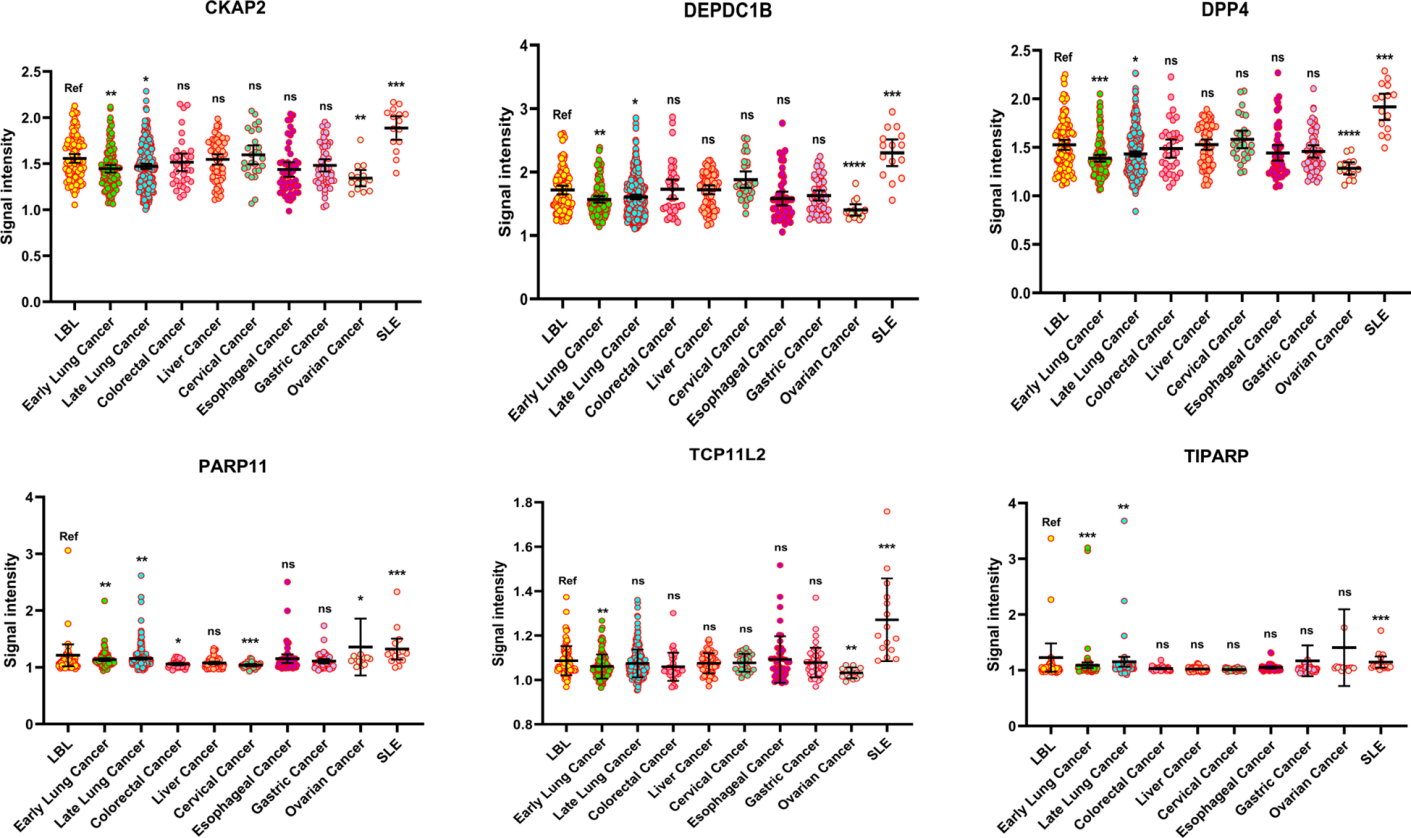
The specificity of the 6 decreased autoantibodies among various cancers. (*P < 0.05, **P < 0.01, ***P < 0.001, ns, no significance).

To evaluate the specificity of the 6 markers in the predictive model in early lung cancers, the serum of patients from 305 lung cancers with advanced stages (stage II~IV), 35 colorectal cancers, 66 liver cancers, 27 cervical cancers, 48 esophageal cancers, 51 gastric cancers, 14 ovarian cancers, and 15 SLEs were collected. The protein levels of LBL were defined as references, and the level in each marker was decreased in early LCs (all *P*<0.01). However, in late LCs, the decline of each marker was less pronounced than that in the early LC group. Compared to the LBL group, the levels of the 6 markers were with no significance in liver cancer, esophageal cancer, and gastric cancer, however, in ovarian cancer, 4 markers (CKAP2, DEPDC1B, DPP4, and TCP11L2) also showed a significant lower level. Moreover, PARP11 seemed to have a lower specificity in lung cancer, as it also decreased significantly in colorectal cancer, and cervical cancer, while elevated in ovarian cancer. In addition, the 6 autoantibodies were all enriched in SLEs (**Figure 5**).

### Decreased autoantibodies are correlated to bone metastasis

**Figure 6:**
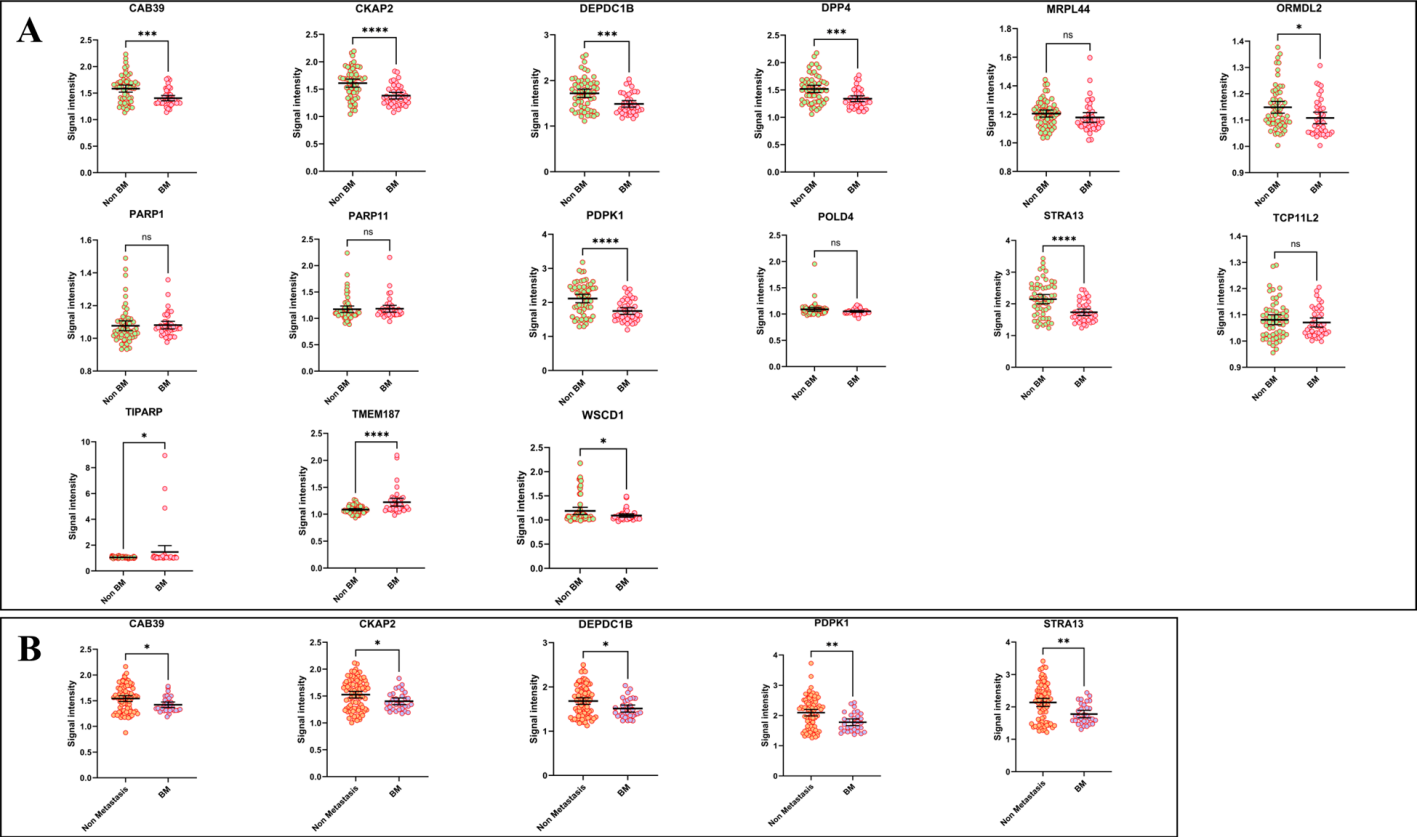
The correlations between decreased autoantibodies and lung cancer bone metastasis. **(A)** comparison of the levels of autoantibodies in LC patients with distant metastasis (non-BM vs BM) **(B)** the levels of autoantibodies between non-metastasis (Stage I~III) samples and BM samples in LUAD. (*P < 0.05, **P < 0.01, ***P < 0.001, ****P < 0.0001, ns, no significance).

There were 99 lung cancer patients with distant metastasis, among them, 41 were with sole bone metastasis (BM group), and 58 were without BM (non-BM group, sole or multiple metastasis of the brain, liver, and adrenal gland). Surprisingly, we found 8 of the candidate autoantibodies (CAB39, CKAP2, DEPDC1B, DPP4, ORMDL2, PDPK1, STRA13, and WSCD1) were significantly decreased in the BM group compared to the non-BM group, while 2 markers (TIPRAP and TMEM187) were adversely increased (**Figure 6A**). However, the 15 autoantibodies showed little differences in the non-metastasis (NM, stage I~III lung cancers) group compared to the non-BM group, or in the NM group compared to the BM group. Besides, subgroup analysis indicated that only in LUAD, 5 biomarkers including CAB39, CKAP2, DEPDC1B, PDPK1, and STRA13, were significant with lower levels in BM patients when compared to NM subjects (**Figure 6B**).

### Follow-up for the postoperative autoantibody levels of early LC patients

**Figure 7:**
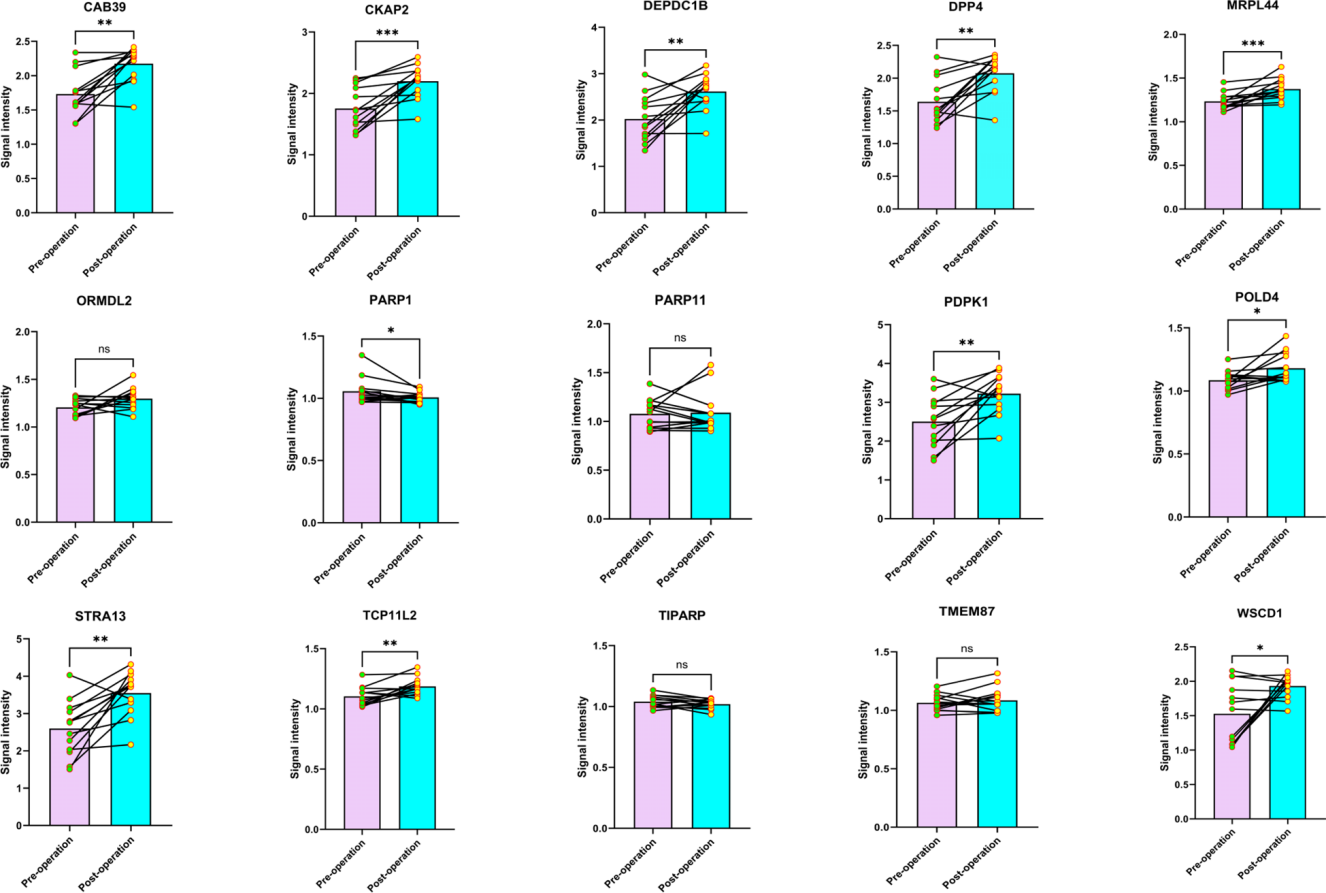
The levels of the autoantibodies in pre-and post-operative samples (*P < 0.05; **P < 0.01; ***P < 0.001, ns, no significance).

As the 15 autoantibodies were strongly correlated to the early-stage LC patients, we next performed follow-ups for 13 patients diagnosed with stage IA NSCLC. The blood samples of the 13 subjects were collected before surgery and 30 days post-operation, respectively, and the levels of the 15 proteins were then measured. It demonstrated that 10 autoantibodies (CAB39, CKAP2, DEPDC1B, DPP4, MRPL44, PDPK1, POLD4, STRA13, TCP11L2, and WSCD1) were elevated as the consequences of resection of the tumor, however, PARP1 was somehow exhibited decreasing tendency in the postoperative period (**Figure 7**). It reversely proved that the decrease of these autoantibodies was attributed to tumor accompanied.

### Proteomic analysis of the CPTAC database

We next employed the CPTAC database to explore the proteins of these corresponding autoantibodies, however, 4 proteins (PARP11, TIPARP, TMEM187, and WSCD1) were not found in the database. The left 11 proteins were respectively evaluated in LUAD and LUSC. Notably, the protein levels of CKAP2, MRPL44, ORMDL2, PARP1, and STRA13 were evaluated in both LUAD and LUSC groups, and for DPP4, the protein levels were increased in LUAD, while down-regulated in LUSC. Additionally, in LUAD, TCP11L2 was the only protein decreased in the tumor samples, and in LUSC, CAB39, DEPDC1B, DPP4, PDPK1, and POLD4 were significantly down-regulated (**Supplementary Figure S2**).

In combination with the clinical data, we then analyzed the prognostic value of each protein. In LUAD, higher levels of DPP4, PARP1, and PDPK1 predicted better prognosis, while the enrichments of CKAP2 and STRA13 were related to poor survival rates (**Supplementary Figure S3A**). In LUSC patients, CAB39 and DEPDC1B were risk proteins as they were correlated with poor prognosis, while POLD4 was identified as a protective protein (**Supplementary Figure S3B**).

To explore the connections between the expression levels of the 11 proteins and the immune cell infiltrations, we conducted a correlation analysis in both the LUAD- and the LUSC-CPTAC groups by applying the EPIC (Estimating the Proportion of Immune and Cancer cells) algorithm. The results indicated that most of the proteins were significantly associated with the proportions of immune cells, especially in the LUSC-CPTAC group (**Supplementary Figure S4**).

### Bioinformatics analysis for the RNA-seq data

To better understand the in-depth mechanisms of the autoantibodies in tumor development and progression, we performed bioinformatics analysis for the mRNAs of the proteins corresponding to the 15 autoantibodies. Since there are great heterogeneities between NSCLCs and SCLCs, we will discuss them separately. When exploring the mRNA levels of NSCLC, The Cancer Genome Atlas (TCGA) dataset containing 110 normal lung samples and 1043 NSCLC tissues was employed. Except for STRA13 and PARP11, other 13 mRNA levels were differently expressed in tumors compared to normal tissues. Among them, the expression of TIPRAP, WSCD1, TCP11L2, DPP4, CAB39 and PDPK1 were down-regulated, while PARP1, DEPDC1B, CKAP2, ORMDL2, MRPL44, POLD4 and TMEM187 were increased in tumor group (**Supplementary Figure S5A**). In evaluating SCLC, we combined the GTEx database (including 288 normal lung samples) and the cBioPortal dataset (81 SCLC RNA-seq data) to make comparisons and the results indicated that 7 genes (TMEM187, WSCD1, MRPL44, PARP11, PARP1, DEPDC1B and CKAP2) were enriched, while 6 genes (POLD4, CAB39, PDPK1, DPP4, TIPRAP, and STRA13) were down-regulated in tumor samples (**Supplementary Figure S5B**). In addition, another GEO dataset (GSE135304) designed to study the whole blood gene expression from patients with malignant or benign nodules was utilized for validation. We found that the mRNA levels of CAB39, PARP1, and PDPK1 were significantly decreased, while STRA13 and WSCD1 were at higher levels in the blood of malignant nodules patients. DPP4 and PARP11 revealed the decreasing tendency in malignant samples, with approximately significant *P* values (**Supplementary Figure S5C**).

We next explored the prognostic values of the mRNAs, and the Kaplan-Meier Plotter online tool was applied for NSCLCs. The best cut-off value to distinguish the low- or high expression of each gene was automatically selected. The results illustrated that each of the 15 genes had favorable prognostic values, and 8 of them (CKAP2, DEPDC1B, MRPL44, ORMDL2, PARP1, POLD4, STRA13, and TCP11L2) were correlated with poorer survival rate, while the other 7 genes (CAB39, DPP4, PARP11, PDPK1, TIPRAP, TMEM187 and WSCD1) were protective factors in NSCLC (**Supplementary Figure S6A**). In SCLCs, we employed the cBioPortal and GSE60052 datasets, and a univariate Cox regression model was conducted. However, as to the limited sample number, none of these genes were found significantly related to the prognosis of SCLCs. It seemed that DEPDC1B predicted poor prognosis since the *P* values approached the threshold of 0.05 in both of the two datasets (*P*=0.058 and *P*=0.065, respectively, **Supplementary Figure S6B**).

We performed further immune infiltration analyses of mRNAs corresponding to the 6 decreased autoantibodies (CKAP2, DEPDC1B, DPP4, PARP11, TCP11L2, and TIPARP) in the predictive model. As there are various algorithms to estimate the infiltration fractions of immune cells, we chose the best method for each kind of immune cell according to the recommendations from Sturm et al. (19). All the correlation analyses were adjusted by tumor purity. In LUAD, CKAP2 AND DEPDC1B were significantly positively associated with CD4+ Th2 cells. The other 4 markers were all positively related to the enrichment of Treg cells. Notably, TCP11L2 was associated with the decrease of CD4+ Th1 cells (**Supplementary Figure S7**). In the LUSC group, we can get a similar conclusion, in addition, DPP4, PARP11, and TCP11L2 were found to be positively correlation with the cancer-associated fibroblasts (CAFs) and endothelial cells (**Supplementary Figure S8**).

## Discussion

In this study, we first explored these decreased autoantibodies in lung cancer and clarified their diagnostic values in pulmonary nodules ≤3cm in diameter. The diagnostic model constructed by the decreased autoantibodies revealed relatively higher sensitivity while lower specificity compared to those models composed of elevated markers in predicting benign or malignant pulmonary nodules. Thus, our study demonstrated that a combination of the decreased autoantibodies could be a new strategy to improve the predictive accuracy for lung cancer. Bone is a common site of lung adenocarcinoma metastasis, and the condition of bone metastasis in the early stage tends to be relatively hidden, but when the symptoms of bone pain are obvious, the cost, as well as the difficulty of treatment, will be increased, while the quality and overall survival time will be significantly reduced. The discovered autoantibodies in this study provided the possibility to detect bone metastasis, as some of them were significantly decreased only in the serum of BM patients. Moreover, 10 decreased markers recovered to a higher level as the tumor had been resected, indicating that these markers could be utilized to evaluate the effectiveness of surgical intervention or monitor the reoccurrence of the tumor. At last, we used public databases to evaluate the mRNAs of the proteins that corresponded to the decreased autoantibodies and found that most of the genes were differentially expressed between normal and tumor samples, meanwhile, all of the genes had favorable prognostic values in NSCLCs, thus supplying novel potential therapeutic targets for lung cancer.

Among the detected 15 autoantibodies, half of the corresponding genes and proteins have yet to be reported in lung cancer, and our study provides evidence for further research on them. CAB39 is a highly conserved protein that binds calcium and can form a heterotrimeric complex with LKB1-STRAD, which is involved in biological activities such as cellular energy metabolism, proliferation, and maintenance of cell polarity (20). CAB39 autoantibody was also found to be decreased in the BM samples, while its gene was down-regulated in both NSCLC and SCLC samples, as well as in the whole blood sample of NSCLCs, however, high levels of CAB39 protein in LUSC were associated with lower survival rates, indicating that CAB39 has complex roles in tumor regulation. A recent study indicated that up-regulated CAB39 could facilitate NSCLC progression (21), whereas the specific mechanisms were not fully cleared. As there is still a lack of evidence of CAB39 on lung cancer, further in-depth studies are needed. Cytoskeleton-associated protein 2 (CKAP2), located in the center of microtubule organization and microtubules, plays an important role in cell mitosis (22). The LUAD bone metastasis patients were accompanied by decreased levels of CKAP2 autoantibody, however, the bioinformatic analysis revealed that CKAP2 was correlated with poorer clinical outcomes in NSCLC, indicating that CKAP2 autoantibody may have positive functions in confronting tumors. Previous studies proved that CKAP2 could promote the progression of triple-negative breast cancer (23), high−grade glioma (24), etc., while there is still a lack of evidence in lung cancer. In our study, according to the CPTAC database, CKAP2 was identified as a risk factor in LUAD but somehow seemed to contribute to a better prognosis in LUSC. Similarly, DEPDC1B autoantibody was also found to decrease in the BM subjects, and it recovered to a higher level after tumor resection. The roles of DEPDC1B have been fully studied, and consistent with our conclusion, DEPDC1B promotes malignant in various types of tumors including lung cancer (25, 26). Our study demonstrated the diagnostic role of DEPDC1B autoantibody in early lung cancer, and there may be some unknown mechanism that prevents the generation of DEPDC1B autoantibody and leads to tumor progression. Based on the bioinformatics analysis, DPP4 (dipeptidyl peptidase 4) was a protective factor, suggesting that the reduction of its autoantibody may be caused by the decreased DPP4 protein in the tumor. DPP4 can hydrolyze many endogenous peptides, resulting in the activation or inactivation of endogenous peptides, and has long attracted attention as a therapeutic target for diabetes mellitus (27). However, the roles of DPP4 on lung cancer remain controversial (28), due to the complex tumor microenvironment, and the variation tendency of its autoantibody may provide new strategies to study its specific functions. MRPL44 belongs to the member of mitochondrial ribosomal proteins, which participate in cell apoptosis (29). Its mRNA and protein were enriched in the tumor samples, and a higher level of the mRNA was related to poorer prognosis, indicating that MRPL44 is a tumor-promoting gene, and its autoantibody may play an anti-tumor role. However, there’s little known about the effects of MRPL44 on malignant disease, and our study provides evidence for further studies. ORMDL2 is localized in the perinuclear endoplasmic reticulum membrane. It has a truncated N-terminal domain and lacks phosphorylated serine residues, which play an important role in sphingolipid biosynthesis and metabolism (30). Until now, there is still a lack of evidence on the functions of ORMDL2 on lung cancer, and we proved that it is an oncogenesis gene, while lower levels of its autoantibody may predict an adverse prognosis. PARP1, PARP11, and TIPRAP(PARP7) are members of the poly (adenosine diphosphate) ribose polymerase family, which is important in DNA damage repair and maintenance of genomic stability (31). PARP1 has been well known as an oncogene, and its inhibitors are widely used in treating various tumors (32) including lung cancer, especially in SCLC (33, 34). In our study, PARP1 is also identified as a tumor promoter gene. However, the mRNA level of PARP1 in whole blood was somehow decreased in the tumor group and the higher protein level of PARP1 in LUAD was correlated to a better prognosis, meanwhile, its autoantibody was surprisingly decreased in the post-operation group. Compared to PARP1, the mechanism of PARP11 in tumor development has not been uncovered. Recent research shows inhibition of PARP11 could improve the efficacy of CAR-T therapy for solid tumors (35). TIPARP plays a key role in innate immune signaling pathways, particularly as a negative regulator of type I interferon antiviral responses (36). In our study, multiple pieces of evidence indicated that it was a tumor suppressor factor, and there is still a need for more in-depth studies. PDPK1 is a phosphorylation-regulated kinase that can be activated by interaction with AKT, thereby promoting activation of the mTOR pathway (37). In our study, PDPK1 is related to better prognosis in lung cancer, especially in LUAD, in addition, its autoantibody can be regarded as a sensitive biomarker in predicting bone metastasis. Nevertheless, studies demonstrated that PDPK1 was a key regulator in promoting lung cancer progression (38, 39), thus these contradictory conclusions require more in-depth mechanistic studies. POLD4 was reported to participate in DNA replication and repair to sustain tumor cell survival (40), and in our study, the transcriptomics analysis indicated that it led to poor prognosis of NSCLC patients, however, the proteomics analysis revealed that it was a protective factor in LUSC. STRA13, also known as BHLHE40 has been reported as a key regulator in immunity during infection, autoimmunity, and inflammatory (41). We found that a low level of STRA13 autoantibody was correlated with bone metastasis, meanwhile, higher expression of STRA13 mRNA predicts longer survival time in NSCLC, indicating that STRA13 may be a tumor suppressor, whereas, in LUAD, a high level of STRA13 protein somehow predicted poor survival rate. Li et al. (42) uncovered that deletion of STRA13 mouse models not only impairs the lethality of CD8+ T cells to tumor cells, but also significantly reduces the formation of CD8+ T cells, and the conclusions are waiting for validation in lung cancer. TCP11L2 has rarely been reported in tumors. In our study, it is strange that the mRNA level was down-regulated in tumors, while its high expression was related to poor survival rate, moreover, the autoantibody of TCP11L2 could increase to a normal level after surgery. The specific role of TMEM187 in cancers has not yet been elucidated, our findings revealed that its mRNA is enriched in tumors, while its higher expression somehow predicted better clinical outcomes. There is still a lack of studies to concern about WSCD1 on lung cancer. Choi et al. (43) reported that WSCD1 was a super-enhancer of glioblastoma. In this study, the autoantibody of WSCD1 was decreased in early LC, and after surgery, the levels of its autoantibody could be recovered, indicating that WSCD1 may well participate in lung cancer development. In NSCLC, the mRNA level of WSCD1 was down-regulated, and combined with the survival analysis, it indicated that WSCD1 owned the ability of antitumor.

The difficulty in the diagnosis of lung cancer is often not with large masses (>3cm); at this stage, patients are frequently accompanied by typical clinical symptoms (e.g. cough, bloody sputum, or chest pain) and the diagnosis can usually be made accurately by the enhanced CT. In patients with nodules ≤3 cm in diameter, the clinical symptoms are commonly not obvious, and the diagnostic value of enhanced CT for such nodules is limited, especially for those with GGNs (44). For this reason, the identification of benign or malignant lung nodules (≤3 cm) and the making of correct clinical decisions based on the results are important to improve the overall survival rate of lung cancer, as surgery can increase the 5-year survival rate of early-stage (stage I) lung cancer to more than 80%, and over 90% for stage IA (45). The currently used 7-TAAbs diagnosis model has a broad application prospect in lung cancer screening (14), showing that the 7-TAAbs model had high specificity but low sensitivity (46). In dealing with early-stage lung cancer, the sensitivity of the 7-TAAbs model was not satisfactory. Xu et al. (47) enrolled 933 subjects, and found that the sensitivity and specificity of the 7-TAAbs model in diagnosing early-stage malignant were 59.7% and 81.1%, compared to our 6 decreased marker model (with 76.19% sensitivity, and 55.74% specificity), and to our 10-markers model (with 80.00% sensitivity, and 87.00% specificity). Another real-world study that recruited 15,430 subjects, demonstrated that the 7-TAABs panel had a sensitivity of 61.5% and a specificity of 88.5% in predicting lung cancer, whereas the sensitivity decreased to only 50.0% for stage I lung cancer (48). However, due to the heterogeneity of our experimental design, most of the antibodies in the 7-TAABs were not detected to be differential in our primary screening phase, and therefore we were unable to validate the diagnostic efficiency of this model in early-stage lung cancer during the validation phase, which is a limitation of our study. In summary, the elevated-autoantibodies model has a relatively high specificity but lower sensitivity, and according to our findings, the decreased-autoantibodies model can improve diagnostic sensitivities. Our study provides new strategies to improve the diagnostic efficacy of early-stage lung cancer.

## Conclusion

Our study demonstrated the diagnostic values of these decreased autoantibodies and combined with those elevated autoantibodies, the 10-markers model can reach a sensitivity of 80.00%, and 87.00% specificity, which are better than the 7-TAABs panel in diagnosed with stage IA lung cancers. Meanwhile, we identified 5 autoantibodies (CAB39, CKAP2, DEPDC1B, PDPK1, STRA13), which can be regarded as biomarkers for bone metastasis in LUAD. In addition, 10 of the decreased autoantibodies returned to a higher level 30 days after tumor resection, indicating that they may be used for evaluating if there are tumor residues after surgery. In conclusion, we discovered the importance of the decreased autoantibodies in lung cancer, providing new thoughts for further studies.

## Data Availability

The original contributions presented in the study are included in the article/**Supplementary Material**. Further inquiries can be directed to the corresponding author.

## Supplementary material

Supplementary Figure 1 The ROC curve for each autoantibody in diagnosing early LCs in the training cohort.

Supplementary Figure 2 The correlations between decreased autoantibodies and lung cancer bone metastasis. **(A)** comparison of the levels of autoantibodies in LC patients with distant metastasis (non-BM vs BM) **(B)** the levels of autoantibodies between non-metastasis (Stage I~III) samples and BM samples in LUAD.

Supplementary Figure 3 The levels of corresponding proteins of the autoantibodies in normal and tumor samples from the CPTAC database.

Supplementary Figure 4 The prognostic values of the corresponding proteins of the autoantibodies. **(A)** in LUAD **(B)** in LUSC.

Supplementary Figure 5 Comparison of the mRNA levels of the 15 markers between normal and tumor samples. **(A)** in the TCGA-NSCLC dataset **(B)** in SCLCs **(C)** in the peripheral blood of NSCLC (green, patients with benign lung nodes; red, patients with malignant lung nodes).

Supplementary Figure 6 The prognostic value of each mRNA in lung cancer. **(A)** the Kaplan-Meier plot for each marker in NSCLCs. **(B)** The forest plot of univariate analysis for each marker in SCLCs.

Supplementary Figure 7 The immune infiltration analyses for the 6 markers in LUAD.

Supplementary Figure 8 The immune infiltration analyses for the 6 markers in LUSC.
