# Supplementary figures and images for: Exploration of the diagnostic and prognostic roles of decreased autoantibodies in lung cancer
Source: Front Immunol. 2025 Jan 30;16:1538071. doi: 10.3389/fimmu.2025.1538071 (PMC11821978; doi:10.3389/fimmu.2025.1538071)

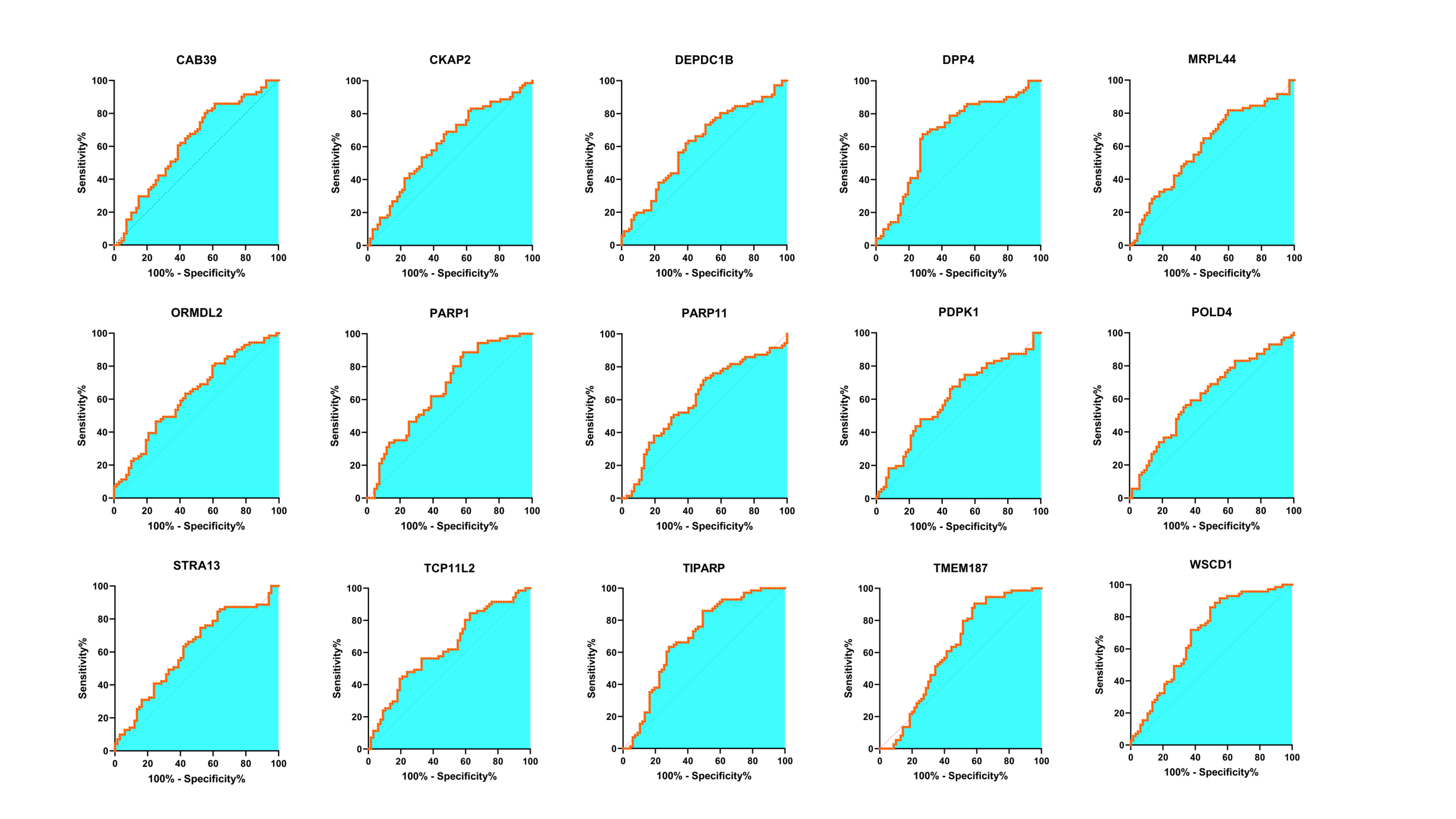

Supplement: Supplementary Figure 1 — The ROC curve for each autoantibody in diagnosing early LCs in the training cohort. [file Image1.tif]

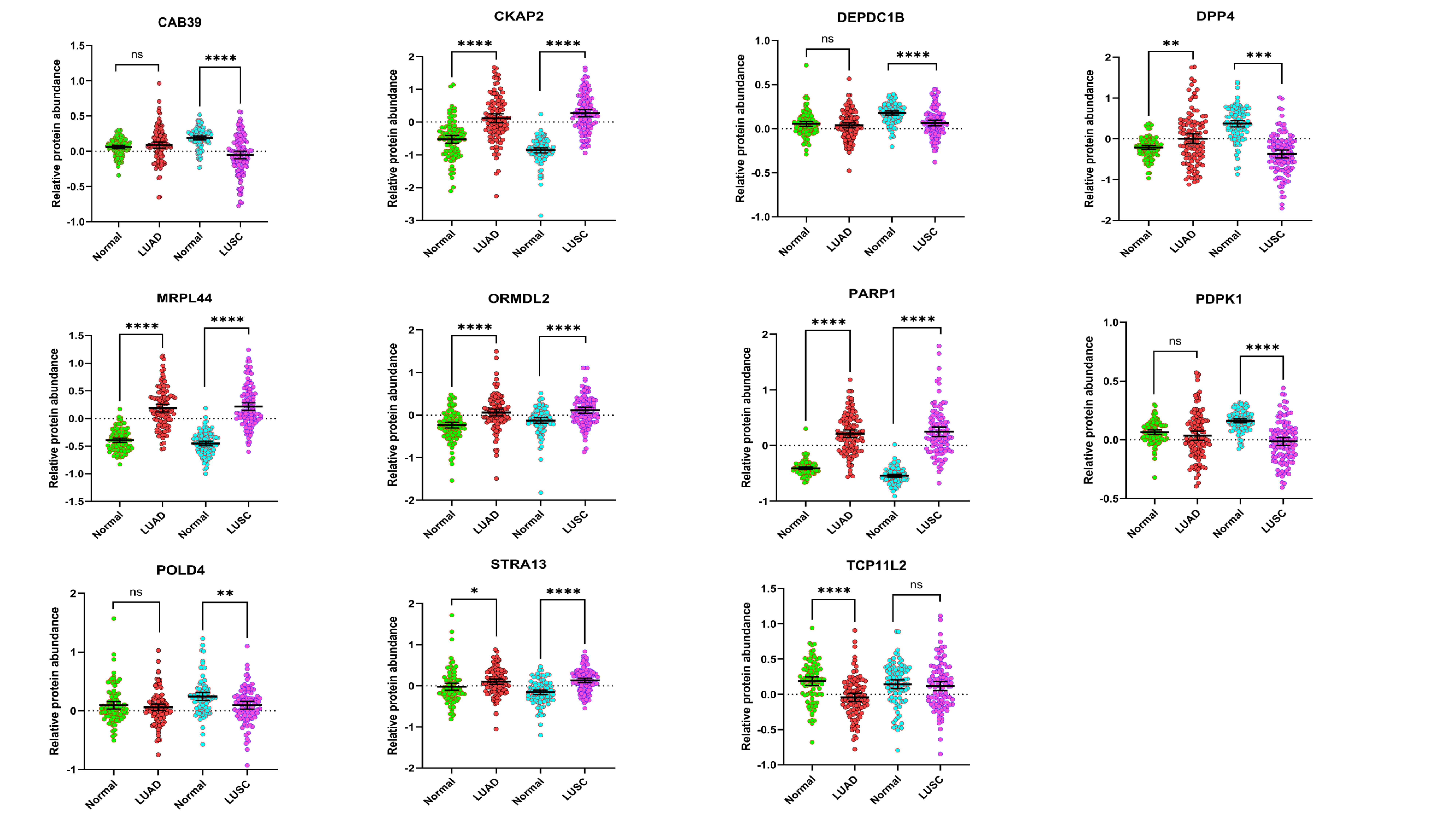

Supplement: Supplementary Figure 2 — The correlations between decreased autoantibodies and lung cancer bone metastasis. (A) comparison of the levels of autoantibodies in LC patients with distant metastasis (non-BM vs BM) (B) the levels of autoantibodies between non-metastasis (Stage I~III) samples and BM samples in LUAD. [file Image2.tif]

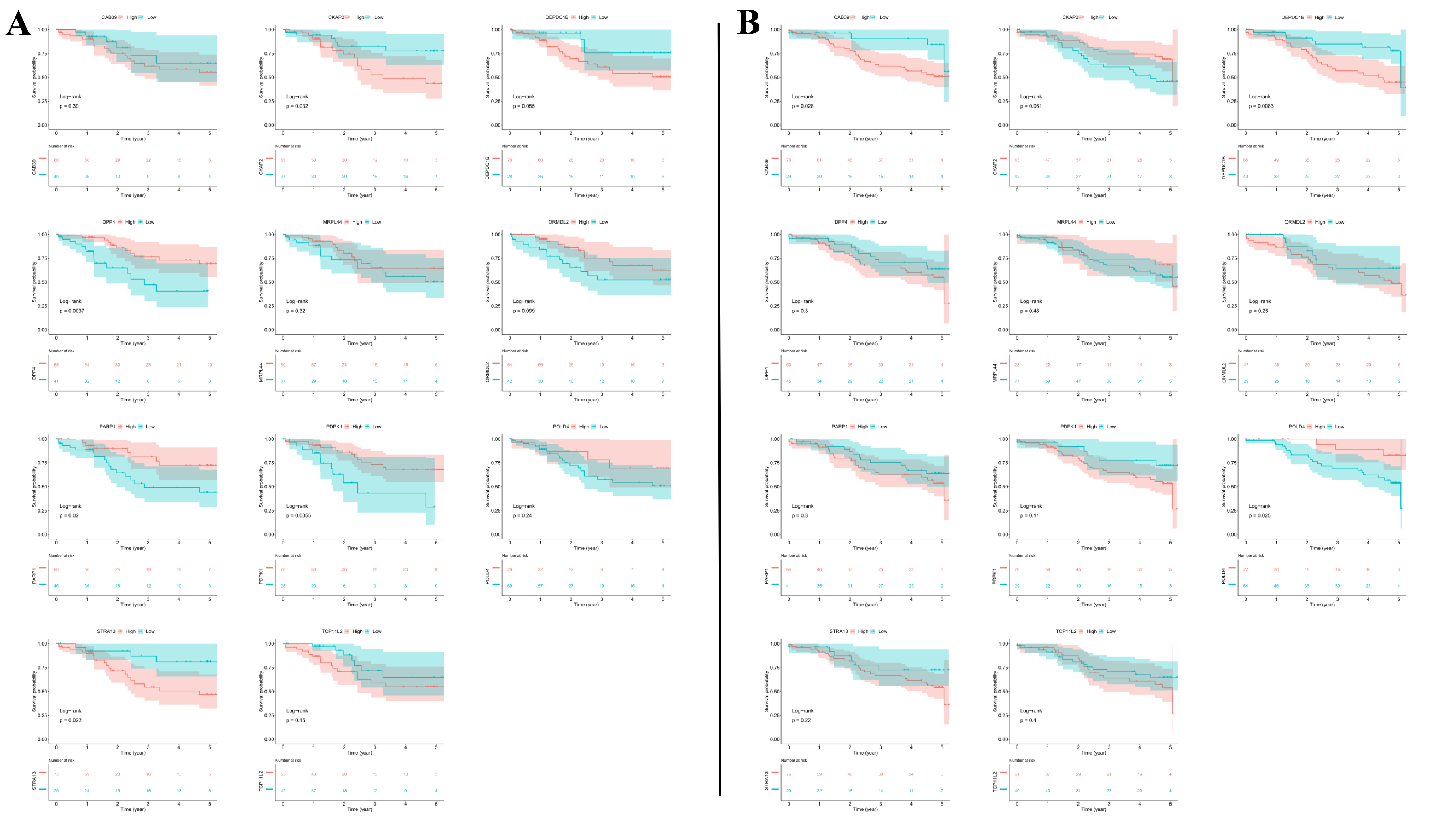

Supplement: Supplementary Figure 3 — The levels of corresponding proteins of the autoantibodies in normal and tumor samples from the CPTAC database. [file Image3.tif]

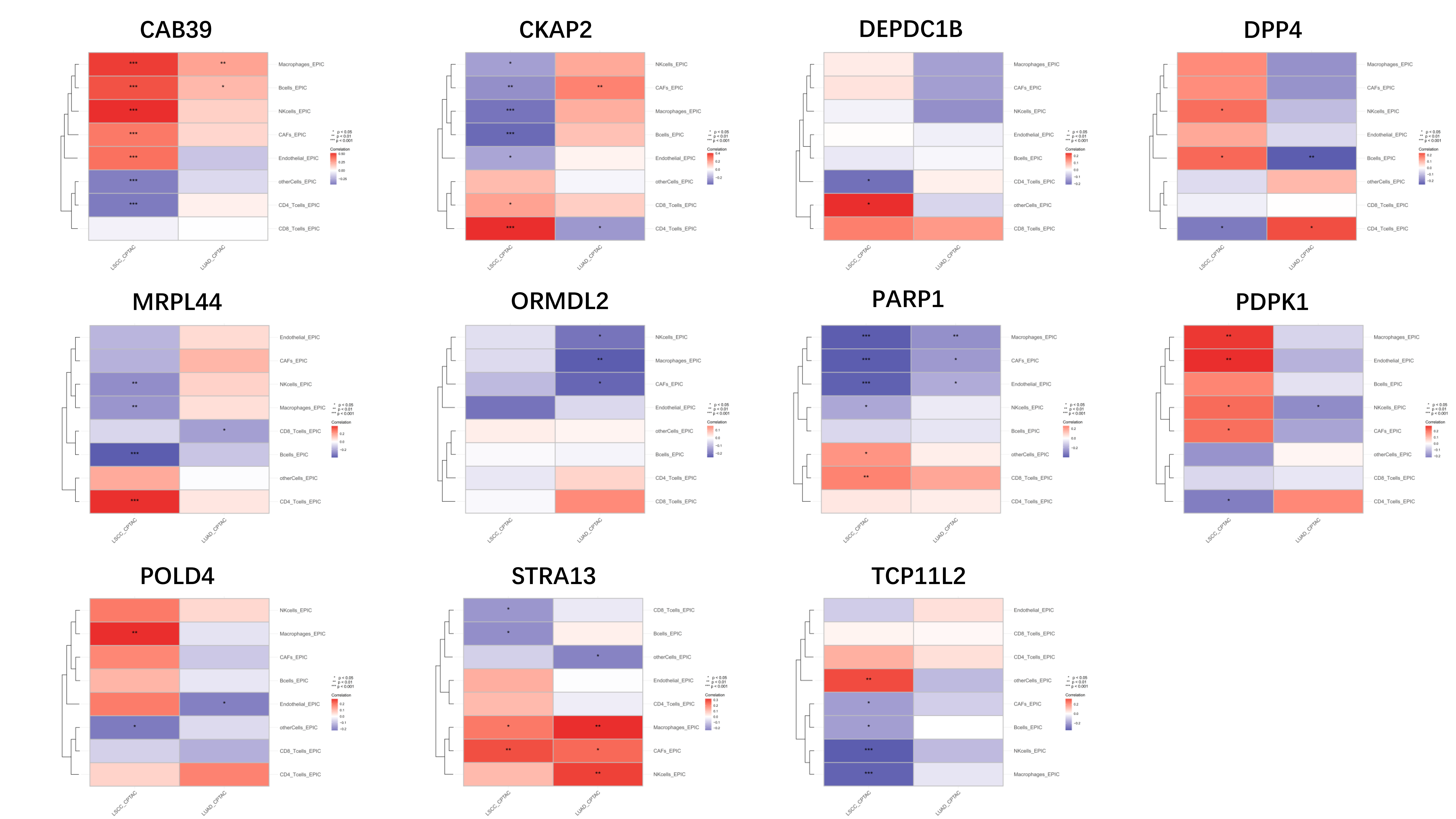

Supplement: Supplementary Figure 4 — The prognostic values of the corresponding proteins of the autoantibodies. (A) in LUAD (B) in LUSC. [file Image4.tif]

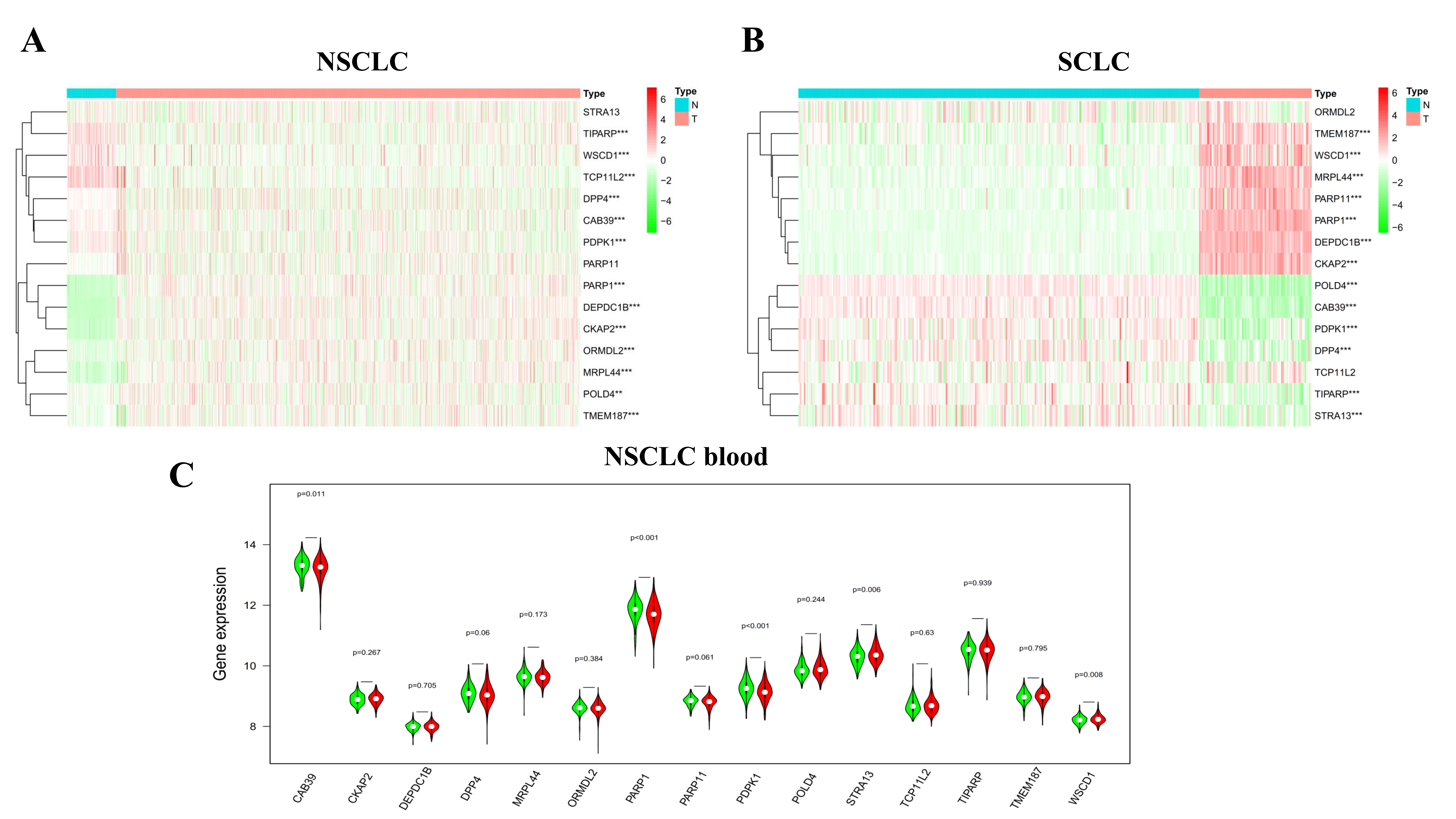

Supplement: Supplementary Figure 5 — Comparison of the mRNA levels of the 15 markers between normal and tumor samples. (A) in the TCGA-NSCLC dataset (B) in SCLCs (C) in the peripheral blood of NSCLC (green, patients with benign lung nodes; red, patients with malignant lung nodes). [file Image5.tif]

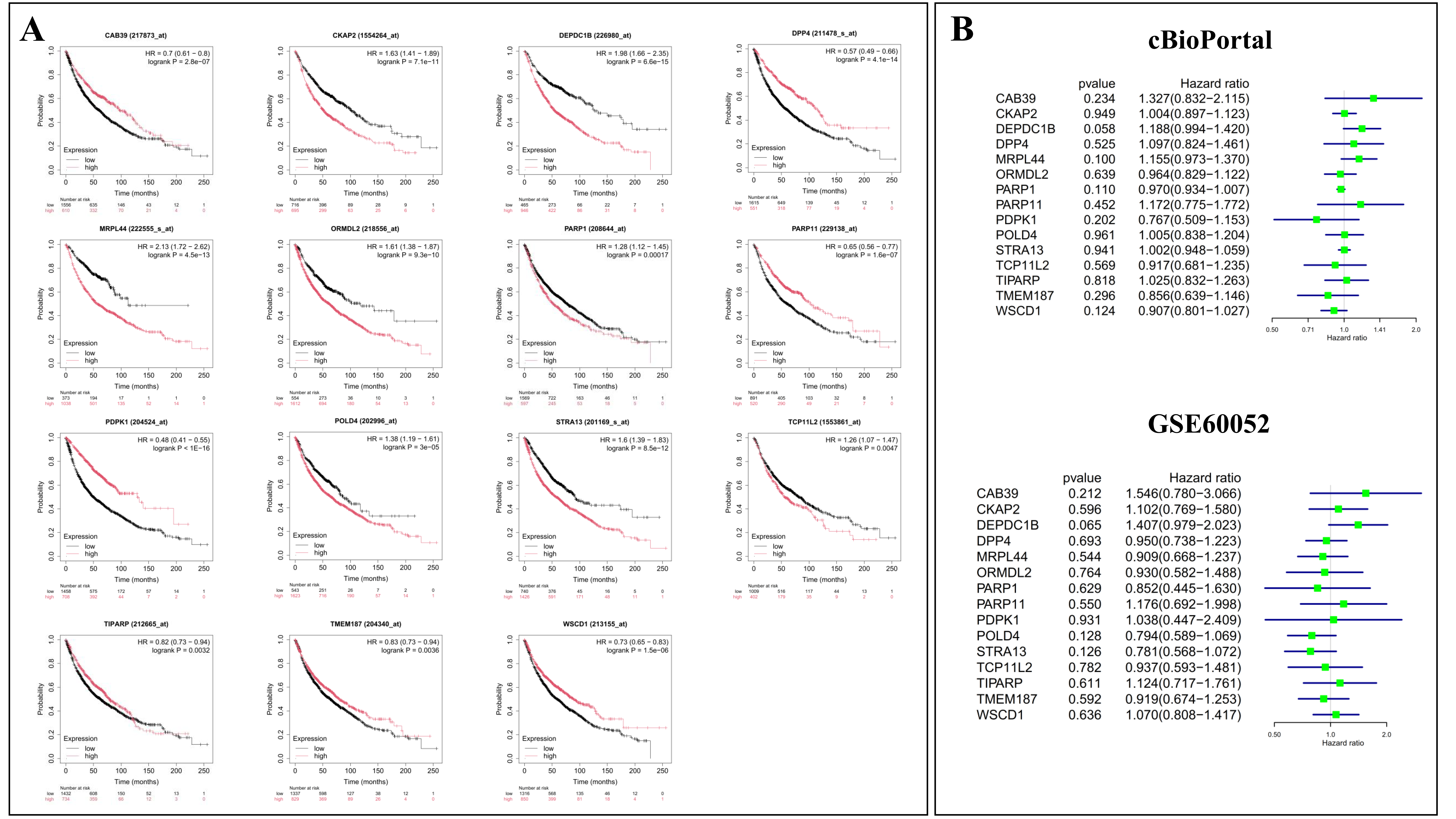

Supplement: Supplementary Figure 6 — The prognostic value of each mRNA in lung cancer. (A) the Kaplan-Meier plot for each marker in NSCLCs. (B) The forest plot of univariate analysis for each marker in SCLCs. [file Image6.tif]

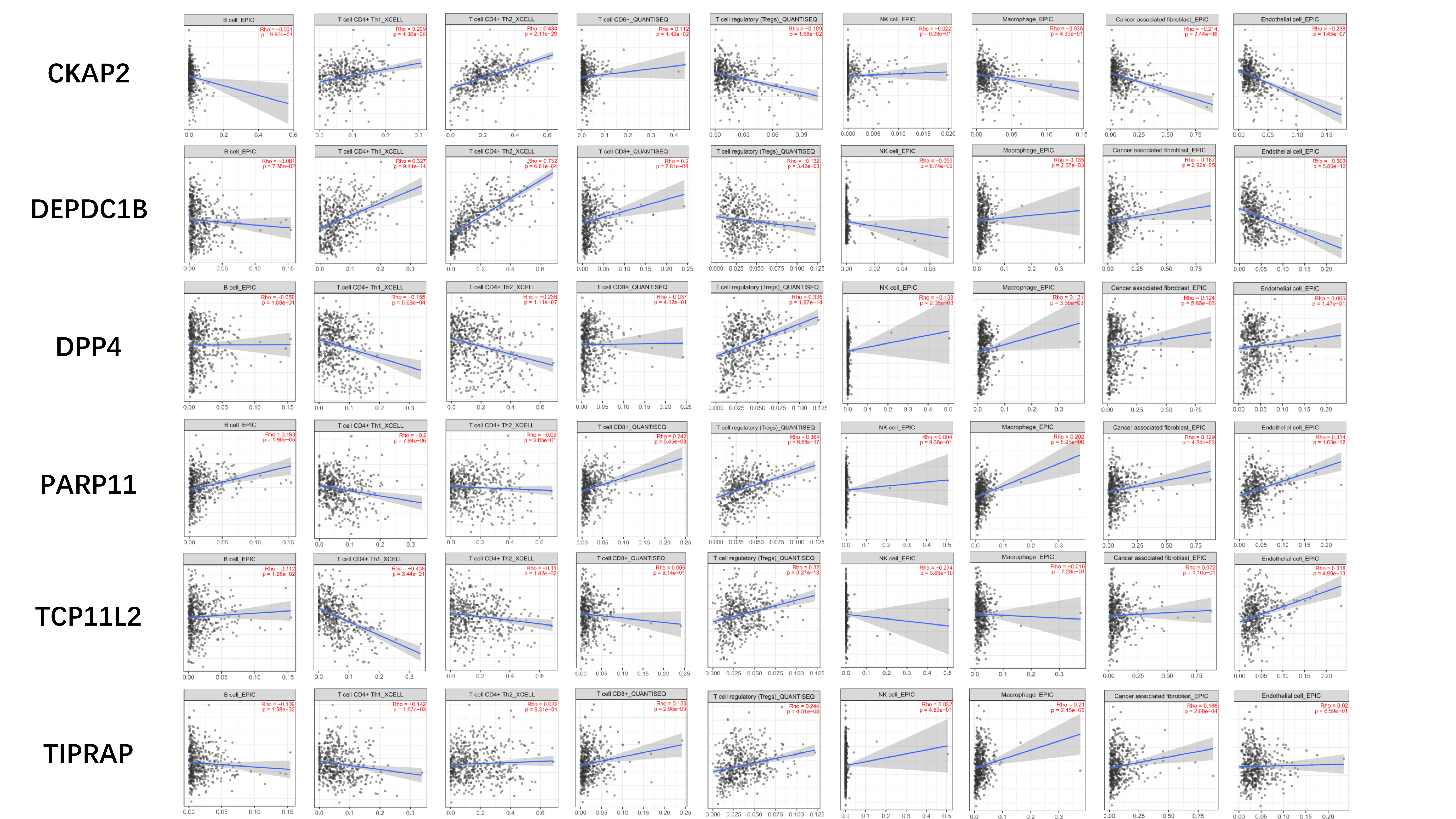

Supplement: Supplementary Figure 7 — The immune infiltration analyses for the 6 markers in LUAD. [file Image7.tif]

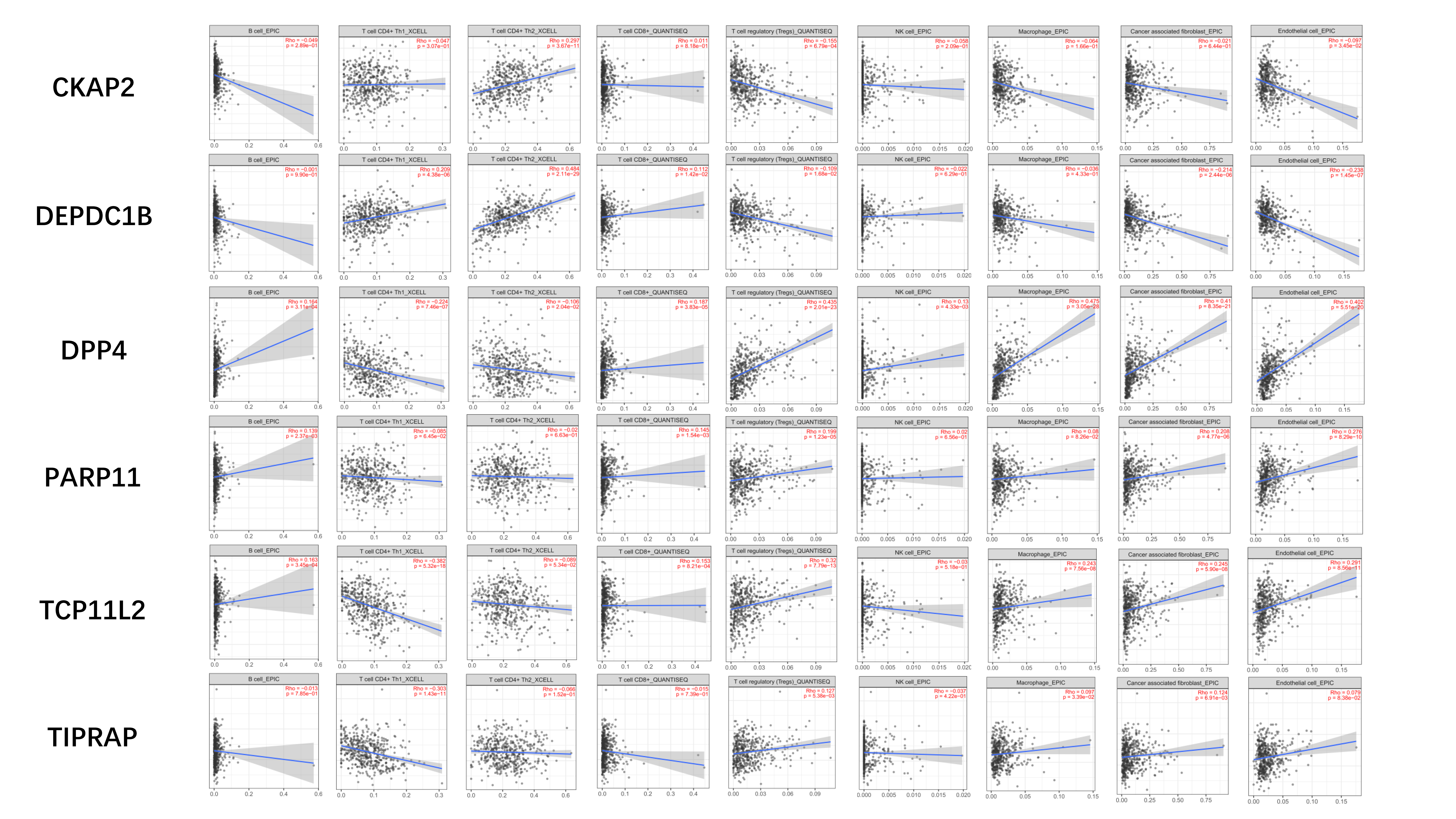

Supplement: Supplementary Figure 8 — The immune infiltration analyses for the 6 markers in LUSC. [file Image8.tif]
